# The Emerging Role of Long Non-coding RNA 01296 in Human Malignancies

**DOI:** 10.2174/0115665240352933250116050706

**Published:** 2025-01-31

**Authors:** Lei Luo, Fan Yang, Xiaoping Fu, Tingting Yu, Wenqian Tang, Juan Xue

**Affiliations:** 1 Department of Health Management Center, Hubei Provincial Hospital of Traditional Chinese Medicine, Wuhan, 430070, P.R. China;; 2 School of Clinical Medical, Hubei University of Chinese Medicine, Wuhan, 443000, P.R. China;; 3 Department of Gastroenterology, Hubei Provincial Hospital of Integrated Chinese and Western Medicine, Wuhan, 430015, P.R. China

**Keywords:** Cancer, long non-coding RNA, LINC01296, molecular mechanisms, therapeutic target, prognostic biomarker

## Abstract

Long non-coding RNAs (lncRNAs) refer to a group of RNA molecules that exceed a length of 200 nucleotides and lack the ability to code proteins. Numerous studies suggest that lncRNAs significantly contribute to the onset and progression of various forms of cancers. A specific lncRNA, known as long non-coding RNA 01296 (LINC01296), is extensively expressed in human malignancies. The level of LINC01296 has been shown to correlate with the progression and prognosis of cancers. Moreover, numerous scientific investigations have provided evidence that the dysregulation of LINC01296 functioning as a competitive endogenous RNA (ceRNA) exerts a profound influence on various aspects of cancer cell behavior, including proliferation, apoptosis, invasion, metastasis, and cell cycle progression, by means of regulating target genes and signaling pathways. An increasing body of data strongly suggests that LINC01296 may serve as a valuable biomarker for predicting cancer prognosis and could represent a promising therapeutic target for cancer intervention. In this comprehensive review, we summarize the recent advancements in our understanding of the role, underlying mechanisms, and clinical significance of LINC01296 in malignant tumors. The findings suggest that LINC01296 may be both a reliable biomarker and a potential therapeutic target for cancers.

## 1 INTRODUCTION

Cancer is a disease that occurs in multiple organs, involves complex biological factors, and is difficult to cure. In recent years, there has been a rapid increase in the incidence and mortality rates of cancer [1]. In the United States, it is estimated that there will be a total of 1,958,310 newly diagnosed cancer cases and 609,820 deaths related to cancer in 2023 [2]. Due to individual variations, tumor heterogeneity, and inherent limitations of current treatments, prognosis remains poor for the majority of patients [3, 4]. To enhance the precision of malignancy treatment and elevate the efficacy of tumor diagnosis and therapy, additional investigation into more promising tumor biomarkers and therapeutic targets is imperative.

LncRNAs refer to a group of RNA molecules that exceed a length of 200 nucleotides and lack the ability to code proteins [5-7]. LncRNAs were initially regarded as a non-functional gene transcription noise due to not having the function of protein-coding [8]. Acting as “RNA sponges” or ceRNAs, they modulate tumor-related biological processes, including cell proliferation, metastasis, and drug resistance. Their roles encompass sequestering microRNAs, binding to RNA-binding proteins, regulating gene transcription, influencing alternative splicing, and affecting protein translation [9, 10]. As lncRNAs are highly specific and easy-to-target therapy, individualized therapy has been used in a variety of cancers [11, 12], and they have been associated with multiple diseases other than cancer, such as respiratory diseases [13], endocrine system diseases [14], and reproductive system diseases [15]. LncRNAs also have high efficiency, specificity, and stability in tissues, which enables their application as a biomarker for cancer detection, prognosis, or therapeutic interventions.

**Fig. (1):**
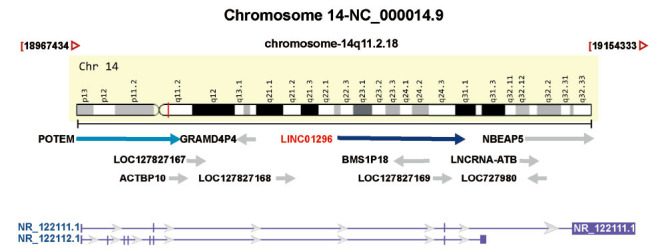
Schematic representation of the LINC01296 transcript and its genomic location (the chromosome map is provided by the Genecard website).

**Fig. (2):**
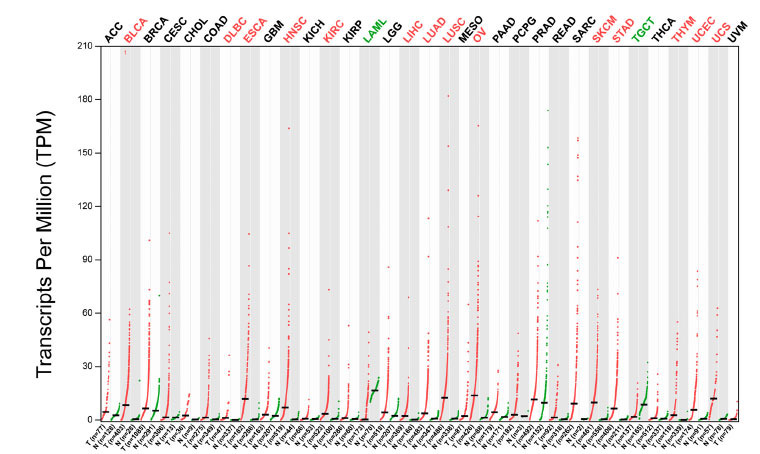
The expression of LINC01296 in various tumors was different from that in normal tissues. Log2FC: 1; Q value: 0.01 [data were obtained from GEPIA (Gene Expression Profiling Interactive Analysis)]. **Abbreviations:** N, Normal tissue, T, tumor tissue. ACC, Adrenocortical carcinoma; BLCA, Bladder cancer; BRCA, Breast cancer; CESC, Cervical squamous cell carcinoma and endocervical adenocarcinoma; CHOL, Cholangiocarcinoma; COAD, Colon adenocarcinoma; DLBC, Lymphoid neoplasm diffuse large B-cell lymphoma; ESCA, Esophageal carcinoma; GBM, Glioblastoma multiforme; HNSC, Head and neck squamous cell carcinoma, KICH, Kidney chromophobe; KIRC, Kidney renal clear cell carcinoma; KIRP, Kidney renal papillary cell carcinoma; LAML, Acute myeloid leukemia; LGG, Brain lower grade glioma; LIHC, Liver hepatocellular carcinoma; LUAD, Lung adenocarcinoma; LUSC, Lung squamous cell carcinoma; MESO, Mesothelioma; OV, Ovarian serous cystadenocarcinoma; PAAD, Pancreatic adenocarcinoma; PCPG, Pheochromocytoma and paraganglioma; PRAD, Prostate adenocarcinoma; READ, Rectum adenocarcinoma; SARC, Sarcoma; SKCM, Skin cutaneous melanoma; STAD, Stomach adenocarcinoma; TGCT, Testicular germ cell tumors; THCA, Thyroid carcinoma; UCEC, Uterine corpus endometrial carcinoma; THYM, Thymoma; UCS, Uterine carcinosarcoma; UVM, Uveal melanoma.

 LINC01296 is encoded by a gene on chromosome 14q11.2.18 with a total length of 4,138 bp [16], and contains two transcripts (https://www.ncbi.nlm.nih.gov/gene/503638) (Fig. **1**). Through GenBank (www.ncbi.nlm.nih.gov/genbank/), we found LINC01296 to be related to cancer and significantly differ among various of cancers, such as liver hepatocellular carcinoma (LIHC) [17], lung adenocarcinoma (LUAD) [18], neuroblastoma [19], and stomach adeno-carcinoma (STAD) [20], by promoting cell proliferation, invasion, and metastasis (Fig. **2**). This review presents a thorough examination of the most recent studies concerning the expression patterns, clinical relevance, molecular mechanisms, and biological roles of LINC01296. Furthermore, here, we address the possible clinical uses of LINC01296 as both a therapeutic target and a diagnostic indicator, and analyze its effectiveness in the treatment outcomes of various tumors. Our objective was to furnish a foundation for subsequent investigations and contribute to a deeper understanding of the regulatory function of LINC01296 in cancers.

## 2 MOLECULAR MECHANISMS OF LINC01296 IN CANCERS

The mechanisms of LINC01296 involved in cancer can be summarized into three types, namely ceRNA function, protein interaction, and pathway interaction.

### 2.1 LINC01296 Functions as a ceRNA

miRNAs are important negative regulators of mRNA expression in the ceRNA network, and the expression of downstream mRNAs is regulated by competitively binding to miRNAs and lncRNAs targeting mRNA as a molecular sponge [21]. As shown in Fig. (**3**), LINC01296 can regulate the expression of its downstream target genes through competitive interaction with miRNA, thus playing a role as an oncogene in different human malignant tumors. In oral squamous cell carcinoma (OSCC), LINC01296, the ceRNA of miR-485-5p, regulates the expression of p21-activating kinase 4 (PAK4); promotes OSCC cell cycle, proliferation, migration, and invasion, and inhibits apoptosis [22]. LINC01296 promotes the progression and metastasis of hepatocellular carcinoma (HCC) cells by targeting miR-122-5p and regulating epithelial-mesenchymal transition (EMT) activity [17]. Evidence from xenograft mouse models suggests that knocking out LINC01296 inhibits the growth of neuroblastoma (NB) tumors *in vivo*. This inhibition is primarily achieved by acting as a sponge for miR-584-5p and miR-34a-5p, thereby modulating tripartite motif-containing 59 (TRIM59) expression [23]. In gastric cancer (GC), LINC01296 competitively binds to miR-122 to up-regulate matrix metalloproteinase-9 (MMP-9) and promote the progression of GC [20]. The deletion of LINC01296 inhibits the proliferation and migration of cholangiocarcinoma (CCA) cells in RBE and CCLP1 cells through sponge adsorption of miR-5905 [24]. As a ceRNA, LINC01296 promotes tumorigenesis by regulating miR-598/Twist1 in non-small cell lung cancer (NSCLC) cells [25]. Hu *et al*. [26] also confirmed that LINC01296 promoted the proliferation and migration of NSCLC cells by inhibiting the expression of miR-5095. Overexpression of LINC01296 promoted the proliferation of cutaneous malignant melanoma (CMM) cells and up-regulation of mitogen-activated protein kinase 1 (MAPK1) through competitive binding with miR-324-3p to promote the malignant progression of CMM [27]. The expression of LINC01296 was increased in both NSCLC and colorectal cancer (CRC) tissues and cells, as shown by Sun *et al*. [28]. This increased expression was associated with metastasis and primarily regulated the miR-141-3p/ZEB1-ZEB2 axis.

### 2.2 Protein Interactions

The function and expression of lncRNAs are regulated by transcription factors [29]. LINC01296 can also directly regulate protein expression to promote tumor progression. Zhang *et al*. [30] confirmed that LINC01296 promoted the proliferation, invasion, and migration of OSCC cells by binding to the SRSF1 protein, promoting the malignant lesions of OSCC. In CRC cells, overexpression of LINC01296 inhibited the expression of p15 and led to the occurrence of CRC [31]. The expression of LINC01296 was found to be heightened in osteosarcoma (OS) cells and tissues, and there existed a positive correlation between the knockdown or overexpression of cyclin D1 and the expression of LINC01296, indicating LINC01296 to exert a carcinogenic effect on OS through cyclin D1 [32].

### 2.3 Pathway Interactions

**Fig. (4):**
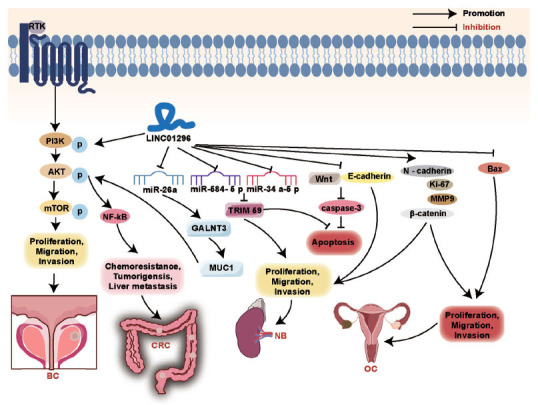
LINC01296 affects three key cell signaling pathways: PI3K/AKT, MAPK/ERK, and Wnt/β-catenin.

LncRNAs participate in the occurrence and development of cancers through various signaling pathways, suggesting that the regulation of related lncRNAs and their signaling pathways may become the target and direction of cancer treatment [33, 34]. Growing evidence suggests that the LINC01296-mediated dysregulation of signaling pathways is central to many different types of cancers (Fig. **4**). PI3K/AKT signaling pathway promotes the occurrence, development, and metastasis of cancer by regulating the proliferation, migration, and apoptosis of cancer cells [35, 36]. Activated PI3K can stimulate the activation of AKT, activate the expression of mTORC1, and promote the proliferation, migration, and invasion of prostate cancer (PC) [37]. In CRC, as the endogenous sponge of miR-26a, LINC01296 regulates the expression of mucin1 (MUC1) under the catalysis of polypeptide N-acetylgalactosaminyltransferase 3 (GALNT3), and then regulates the activity of PI3K/AKT pathway, thus promoting the progress of CRC [38]. Disorders of the MAPK/ERK signaling pathway can be observed in many types of cancers, including OSCC. LINC00319 regulates the expression of PAK4 through sponge miR-485-5p-mediated expression of p-MEK and p-extracellular signal-regulated kinase (p-ERK), thus affecting the progression of OSCC [22]. The Wnt/β-catenin signaling cascade is a highly conserved pathway whose abnormal activity significantly drives cancer stem cell renewal, cell proliferation, and differentiation, thus playing a key role in tumorigenesis and treatment response [39, 40]. In NB cells, down-regulation of LINC01296 could inhibit NB cell proliferation, migration, and invasion, and induce apoptosis by decreasing the protein levels of Wnt1, β-catenin, Ki67, and N-cadherin, and increasing the protein expressions of E-cadherin and cleaved caspase-3 [41]. Lin *et al*. [42] found that knocking down LINC01296 expression could reduce the resistance of SKOV3/DDP cells to cisplatin by inhibiting the Wnt/β-catenin pathway.

## 3 FUNCTIONAL ROLES OF LINC01296 IN DIFFERENT CANCERS

Numerous research studies have shown a signi-ficant correlation between the abnormal expression of LINC01296 and the unfavorable prognosis of patients across multiple cancer types (Table **1**). Additionally, the role and mechanism of LINC01296 in cancer pathogenesis are summarized in Tables **2** and **3**.

### 3.1 Ovarian Cancer

OC is a common malignant tumor in gynecology, which has the characteristics of high incidence, high mortality, high metastasis, and high invasion [43, 44]. As a result of the absence of early specific symptoms and diagnostic markers, most OC patients are typically diagnosed at the middle or advanced stages, leading to a 5-year survival rate of only approximately 30% [45]. Thus, to enhance patient survival rates, it is imperative to identify novel biomarkers for the early detection of OC. Analysis of data retrieved from the GSE14407 and GSE54388 databases revealed a significant upregulation in the expression of LINC01296 in both OC tissues and cells, and patients with high levels of LINC01296 expression in OC tissues displayed a shorter overall survival rate [46]. Suppression of LINC01296 has been demonstrated to hinder OC cell proliferation, migration, and invasion, while promoting apoptosis. Further studies have found the inhibition of miR-29c-3p expression to intensify OC cells’ proliferation, migration, and invasion, underscoring the role of LINC01296 in the initiation and progression of OC through miR-29c-3p regulation [47]. Cisplatin, a commonly used antitumor drug, plays an important role in the chemotherapy of OC. Cisplatin resistance is an important factor leading to the recurrence of OC. The expression of LINC01296 was significantly increased in cisplatin-resistant OC cell lines, OVCAR-3 cells, and LINC01296 knockdown could enhance cell sensitivity and reduce drug resistance index [48]. Further study showed that LINC01296 reduced the resistance of OC cells to cisplatin by inhibiting the Wnt/β-catenin pathway [42]. Consequently, LINC01296 holds promising potential as a novel biomarker and therapeutic target for both the diagnosis and treatment of OC.

### 3.2 HCC

HCC is the leading form of primary liver cancer, comprising approximately 80%-85% of all liver cancer cases. Epidemiological data show that HCC ranks fifth by incidence and second by cancer-related mortality globally [49]. To enhance the diagnosis and treatment of HCC patients, it is imperative to delve into the molecular mechanism of HCC and discover early tumor markers. Research has unveiled that LINC01296 is significantly increased in HCC, and is closely related to alpha-fetoprotein level, tumor size, and tumor node metastases (TNM) stage. The higher the expression of LINC01296 in HCC, the lower the overall survival rate and the worse the prognosis [14]. Functional experiments showed that LINC01296 could promote the migration and invasion of HCC cells by promoting EMT [14]. In terms of mechanism, inhibition of LINC01296 inhibits the growth of HCC *in vivo*, inhibits the proliferation, migration, and invasion of HCC cells *in vitro*, and plays a role in the occurrence and development of HCC by regulating the miR-26a/PTEN signaling pathway [50].

### 3.3 NB

NB is the most common extracranial solid tumor in children, with 90% of cases occurring in children under the age of 10 years [51, 52]. The differential expression of lncRNA in early NB and advanced NB was analyzed by gene chip technology; the analysis revealed an increase in the expression of LINC01296 in advanced NB. Moreover, patients exhibiting higher levels of LINC01296 expression demonstrated a shorter overall survival time, indicating that elevated LINC01296 expression can serve as a prognostic marker for NB patients, predicting invasiveness and poor prognosis [53]. Further experiments *in vitro* showed that silencing LINC01296 could inhibit the proliferation and migration of NB cells while promoting apoptosis. Similarly, the knockdown of LINC01296 hindered xenograft growth in *in vivo* models [54]. Notably, NB cells and tissues displayed a down-regulation of miR-584-5p, whereas LINC01296 exhibited significant up-regulation in NB tissues and cells. Further investigations have found LINC01296 to function as a sponge for miR-584-5p, thereby promoting the expression of TRIM59 and facilitating the progression of NB. These findings provide a promising therapeutic target for NB treatment [23].

### 3.4 Esophageal Squamous Cell Carcinoma (ESCC)

As the primary histopathological subtype of esophageal cancer, ESCC has the characteristics of high incidence and high recurrence rate [55]. Despite the continuous development of treatments (such as surgery, radiotherapy, chemotherapy, and targeted therapy), the 5-year survival rate remains notably low [56]. Quantitative reverse transcriptase PCR (qRT-PCR) analysis revealed a substantial increase in LINC01296 mRNA levels in ESCC cell lines and tissues [57]. Moreover, the high expression of LINC01296 correlated with ESCC tumor differentiation, lymph node metastasis, TNM stage, and distant metastasis. *In vitro*, it has been shown that knocking down LINC01296 inhibits the proliferation, migration, and invasion of ESCC cells, and promotes the expression of Kruppel-like factor 2 (KLF2). *In vivo,* sh-LINC01296 or lv-control was subcutaneously inoculated into mice. After 4 weeks, the sh-LINC01296 group displayed significantly reduced xenograft growth rate and size, along with remarkable up-regulation of KLF2 protein, indicating that LINC01296, as an oncogene, contributes to ESCC progression by regulating KLF2 protein expression, thereby offering potential opportunities for ESCC treatment [58].

### 3.5 CRC

Ranked as the world's third most prevalent malignant tumor and the second leading cause of cancer-related death, CRC brings a huge economic burden to families and society [59, 60]. Elevated expression of LINC01296 expression has been shown in CRC patients, correlating with reduced overall survival rates, advanced tumor stage, lymph node metastasis, and distant metastasis [31]. Chemotherapy is the main treatment for CRC, and 5-fluorouracil is one of the main chemotherapeutic drugs. 5-fluorouracil chemotherapy resistance often leads to treatment failure in patients with CRC [61-63]. Further studies on the molecular mechanism have revealed the role of LINC01296 as an endogenous miR-26a sponge that influences MUC1 expression, catalyzed by GALNT3. Moreover, this interaction modulates the activity of the PI3K/AKT pathway, thereby contributing to CRC progression [38]. Other studies have also confirmed that LINC01296 is highly expressed in CRC and positively correlates with poor prognosis, which is mainly involved in the occurrence and development of CRC through sponging miR-141-3p and regulating ZEB1-ZEB2 [28, 64]. Consequently, a comprehensive exploration of the mechanisms underlying LINC01296 bears immense importance toward enhancing the diagnosis and treatment of CRC in future endeavors.

### 3.6 Bladder Cancer (BC)

Although surgery, radiotherapy, and chemotherapy have been extensively utilized in the treatment of BC, patient prognosis varies and remains dismal, particularly for those with highly invasive forms [65, 66]. Hence, it is imperative to discover novel therapeutic targets that are effective, as well as reliable biomarkers. The analysis of LINC01296 expression using an interactive gene expression profile database revealed a significant upregulation of LINC01296 in BC tissues. Subsequently, qRT-PCR was employed to confirm the expression of LINC01296 in both BC tissues and cells, thereby establishing a consistent conclusion [67]. Elevated expression levels of LINC01296 are associated with poor prognosis and short survival time. Silencing LINC01296 could decrease the viability and migration ability of BC cells *in vitro* [68].

### 3.7 Lung Cancer

Lung cancer, a malignant tumor with a high incidence worldwide, has become a serious public health problem and poses a major threat to human well-being. Statistics indicate that in 2022, the United States reported approximately 237,000 new cases of lung cancer, ranking it second among all malignancies [69]. In China, there were 871,000 new cases of lung cancer in the same year, making it the most prevalent malignant tumor in the country [69]. Therefore, the discovery of biomarkers for early detection and treatment of lung cancer holds immense importance. The two main types of lung cancer are small cell lung cancer (SCLC) and NSCLC, with NSCLC constituting approximately 85% of all lung cancer cases [70].

The expression level of LINC01296 in NSCLC is significantly higher than that in adjacent non-tumor tissues and overexpression of LINC01296 promotes the proliferation and migration of NSCLC cells [26]. Knocking down LINC01296 in mouse xenotrans-plantation could inhibit tumor growth and metastasis, mainly through the reduction of ATG2B expression by sponging miR-143-3p [18]. Similarly, LINC01296 also acts as a tumor promoter by sponging miR-1255b-5p to promote the development of NSCLC [71]. EMT mediates metastasis and recurrence in NSCLC. Compared to para-cancer tissues, high expression of LINC01296 in NSCLC tissues down-regulated the mRNA and protein expression of E-cadherin and promoted the mRNA and protein expression of vimentin and MMP-9, indicating LINC01296 to be involved in regulating the EMT process and promoting invasion and metastasis of NSCLC [72]. Xu *et al*. [25] confirmed that increased levels of LINC01296 positively correlated with the poor prognosis of NSCLC patients, whereas knocking down LINC01296 inhibited NSCLC cell proliferation and promoted apoptosis.

### 3.8 OSCC

OSCC is the most common form of oral cancer [73]. According to statistical data, over 500,000 fresh cases of OSCC are reported annually, resulting in more than 140,000 deaths [74]. Despite significant advancements in surgical resection, radiotherapy, and chemotherapy, the overall prognosis of OSCC patients remains largely unchanged [75]. Therefore, a thorough investigation of the molecular mechanism behind OSCC is essential for the discovery of novel and effective treatment approa-ches. The expression of LINC01296 in OSCC tissues is significantly increased, and the overexpression of LINC01296 promotes the proliferation, migration, and invasion of OSCC cells, and promotes the formation of xenografts [30, 76]. Zhang *et al*. [22] found that LINC01296 regulated PAK4 expression through the spongification of miR-485-5p, activated MAPK/ERK signaling pathway, promoted OSCC cell cycle, proliferation, migration, and invasion, and inhibited apoptosis. LINC01296 is overexpressed in the TCGA-OSCC database, and the high expression of LINC01296 is closely related to poor prognosis [77]. In conclusion, the high expression of LINC01296 in OSCC tissues and cells indicates its potential as a promising therapeutic target for OSCC.

### 3.9 Other Tumors

The qRT-PCR findings have demonstrated a significant increase in the expression of LINC01296 in breast cancer (BRCA) tissues compared to adjacent tissues. Moreover, the enhanced expression of LINC01296 positively correlated with BRCA tumor size, lymph node metastasis, and TNM stage. Further survival analysis revealed that BRCA patients with high LINC01296 expression had a significantly lower 5-year survival rate compared to those with low LINC01296 expression [78]. A study based on head and neck squamous cell carcinoma (HNSCC) found the expression of LINC01296 to be increased in HNSCC cells, and overexpression of LINC01296 promoted the migration and invasion of tumor cells, suggesting that LINC01296 may be a potential diagnostic and prognostic indicator of HNSCC [79]. Qin *et al*. [20] also confirmed the high expression of LINC01296 in GC and its association with a poor prognosis. Suppression of LINC01296 effectively inhibited the proliferation, migration, and invasion of GC cells, promoted apoptosis, and promoted the expression of MMP-9 mainly through sponging miR-122 to participate in the occurrence of GC. Yu *et al*. [32] studied the mechanism of LINC01296 involved in OS and found that LINC01296 promotes the progression of OS by regulating the proliferation, migration, and apoptosis of OS cells through cyclin D1. CCA is an extremely aggressive and metastatic cancer with a difficult diagnosis and high mortality. In CCA, LINC01296 directly controls the expression of MYCN by acting with miR-5095 to facilitate the migration and invasion of CCA cells [24]. Other investigations have reported that LINC01296 is overexpressed in PC [37], CMM [27], pancreatic ductal adenocarcinoma (PDAC) [80], and clear cell renal cell carcinoma (ccRCC) [81], which contributes to the initiation and progression of cancers. Although information about these other cancer types is relatively limited, our review underscores the strong association between elevated LINC01296 expression and unfavorable prognosis in related cancers. Therefore, LINC01296 holds promising potential as a novel diagnostic and therapeutic target.

## 4 THE POTENTIAL CLINICAL APPLICATIONS OF LINC01296 IN CANCERS

The identification of molecular biomarkers of cancer is of great significance for early prevention, treatment guidance, and prognosis prediction [82]. The current biomarkers for cancer diagnosis and prognosis assessment, such as carcinoembryonic antigen (CEA) and cancer antigen 19-9 (CA19-9), are hindered by their unsatisfactory specificity and sensitivity [83-85]. A large number of studies have shown that lncRNAs are widely present in human cancer tissues, blood (or plasma), and urine [86, 87]. qRT-PCR is often used to detect circulating lncRNA levels. In recent years, lncRNAs have become a new hotspot biomarker for cancer diagnosis, prognosis, and treatment because of their inherent detectability and specificity [88, 89]. Likewise, experiments conducted both *in vitro* and *in vivo* have demonstrated the significant involvement of LINC01296 in the progression of various cancer types and made it a promising molecular target for therapeutic interventions with clinical relevance.

### 4.1 LINC01296 as a Potential Diagnostic Biomarker in Cancers

Employing cutting-edge technology, we identified the presence of LINC01296 in clinical tissue samples and observed its expression to be limited in normal tissues, yet significantly elevated in various cancer types, including HCC [17], NB [23], CMM [27], CRC [38], ESCC [57], etc., which makes LINC01296 a reliable biomarker for cancer diagnosis. Notably, a marked rise in LINC01296 levels was noted in tissue specimens from OC patients, distinguishing them from healthy individuals [46]. The robust stability of lncRNAs in circulation, coupled with their resistance to enzymatic degradation, renders them more reliable biomarkers compared to other nucleic acids. Nevertheless, existing findings mainly focus on tissue-level detection of abnormal LINC01296 expression. Future investigations should aim to analyze LINC01296 expression in bodily fluids, like serum, plasma, and peripheral blood cells, for potential use in diagnosing malignant tumors. Moreover, combining LINC012*9*6 with established cancer biomarkers holds promise in enhancing the accuracy and specificity of cancer diagnosis.

### 4.2 LINC01296 as a Therapeutic Target in Cancers

Given the marked difference in LINC01296 expression between normal and cancerous tissues, gene knockout of LINC01296 could potentially slow tumor progression and offer a novel treatment avenue. Zhang *et al*. [22] highlighted the role of LINC01296 as a ceRNA, which modulates PAK4 expression by seques-tering miR-485-5p, consequently facilitating OSCC progression. Hence, targeting the LINC01296/miR-485-5p/PAK4 axis could be a viable therapeutic target for OSCC. In xenograft mice, silencing LINC01296 markedly impeded xenograft growth [24, 25]. Resistance to chemotherapy represents a significant hurdle in tumor treatment efficacy. Lowering LINC01296 levels can diminish the expression of miR-143-3p's target gene, ATG2B, thereby enhancing paclitaxel sensitivity in NSCLC and offering a promising approach for NSCLC chemotherapy in clinical settings [18]. In CRC tissues, LINC01296 expression was elevated compared to normal tissues and correlated with tumor stage, lymph node metastasis, and distant metastasis. Further use of antisense oligonucleotides to knock down LINC01296 *in vitro* can inhibit the proliferation of CRC cells and promote their apoptosis [31]. These findings may provide a basis for future targeted therapy of CRC. As an emerging lncRNA, research on LINC01296 remains in its preclinical phase, necessitating further studies with larger sample sizes and multicenter participation. We are optimistic that as research progresses, LINC01296 will become a key therapeutic strategy in clinical cancer treatment.

### 4.3 LINC01296 as a Prognostic Marker in Cancers

The monitoring of cancer prognosis is a crucial strategy in reducing cancer-related deaths. High levels of LINC01296 expression have been linked to poor prognoses in multiple cancer types and may be used as a reliable biomarker for different cancers [90]. QRT-PCR analysis of 55 cases of BC and normal tissues adjacent to cancer showed that the expression of LINC01296 was significantly increased in BC tissues, and the elevated level of LINC01296 was positively correlated with tumor stage, lymph node metastasis, and tumor pathological grade [78]. A meta-analysis conducted by Feng *et al*. of 9 studies indicated that increased LINC01296 expression could predict overall survival in OS patients. Furthermore, this elevated expression correlated significantly with clinical stage, lymph node metastasis, tumor size, and differentiation in OS patients, suggesting LINC01296 as a prognostic biomarker [87]. Similar associations were observed in OSCC [22], CRC [64], and NSCLC [91], where elevated expression correlated with higher overall survival rates. In PDAC, increased LINC01296 expres-sion in tissues and cell lines was related to tumor stage and lymph node metastasis, serving as an independent prognostic factor in PDAC patients [80]. In addition, LINC01296 independently predicted out-comes in PC patients [37]. Overall, monitoring LINC01296 levels dynamically may aid in evaluating malignant tumor severity and tracking tumor progression.

## CONCLUSION AND FUTURE PERSPECTIVE

LncRNAs have shown immense potential as emerging biomarkers in cancer research. LINC01296 is considered to be a tumor-promoting gene for almost all types of cancer, such as NSCLC, GC, OC, glioma, OS, and HCC. LINC01296 plays crucial roles in different biological reactions in human malignancies, including proliferation, apoptosis, invasion, migration, cell cycle arrest, and EMT. Additionally, LINC01296 expression is clinically significant in cancer, correlating with advanced TNM stage, larger tumor size, more lymph node metastasis, tumor invasion, and negative prognosis. Mechanistically, LINC01296 functions as a ceRNA and regulates target genes and related signaling pathways (such as PI3K/AKT, MAPK/ERK, and Wnt/β-catenin signaling pathway) by sponging miRNAs, thereby affecting the biological phenotype of tumor cells.

Although numerous studies have elucidated the biological roles and molecular mechanisms of LINC01296 in a variety of cancers, the range of cancer types studied is limited, and the mechanisms and biological functions of LINC01296 in different cancer types are not yet comprehensive. Future emphasis should be placed on unexplored mechanisms of LINC01296, such as immunotherapy. LncRNAs are involved in the immune escape of cancer cells and the regulation of tumor microenvironment. Targeting lncRNAs in tumor or immune cells can enhance anti-tumor immune response and improve the effect of tumor immunotherapy [92, 93]. Understanding the functional role of LINC01296 in cancer and the immune system and its role as a target for cancer immuno-therapy can provide valuable insights for innovative diagnostic and therapeutic approaches. In addition, LINC01296 is widely present in body fluids, and it is imperative to develop reliable methods for stable and efficient detection of LINC01296 in body fluids, such as nanoplasma probes, qRT-PCR, and microarray assays [94, 95]. Additionally, most studies rely on *in vitro* models or xenotransplantation of mice and may not accurately reflect the clinical situation. Whether LINC01296 has corresponding anti-cancer effects in humans remains to be further studied. It is essential to promote the clinical application of LINC01296 and expand the sample size and verify it in a wider population.

The therapeutic potential of LINC01296 is great, but it also faces challenges in terms of stability, off-target effects, and efficient delivery. Advances in nano-technology and synthetic biology show promise to overcome these barriers, and the integration of multi-omics data, including genomics, transcriptomics, epigenomics, and proteomic profiles, can provide a more comprehensive knowledge of the molecular pathways by which LINC01296 occurs and develops in cancer. In addition, computational biology and artificial intelligence are transforming our understanding of lncRNA interactions, guiding the development of more effective treatments [96]. Targeting LINC01296 with specific inhibitors is a promising method for cancer diagnosis and prognosis. Various strategies, including RNA interference [97], antisense oligonucleotides [98], CRISPR-Cas technology [99], and small molecule inhibitors [100], can be used to target LINC01296 for therapy, enhancing the effectiveness of existing therapies. For example, CRISPR-Cas platforms can be used to programmatically knock down cancer-causing lncRNAs and reduce off-target effects, while overexpression vectors can be used to promote tumor-inhibiting lncRNA expression [101]. These techno-logical advances provide a solid foundation for the potential of LINC01296 as a biomarker and therapeutic target. The search for LINC01296 as a therapeutic target is still in the early stages of development. Future research should focus on developing an effective delivery system for LINC01296 targeted therapy and investigating its efficacy in clinical trials.

This article has reviewed the function and regulatory mechanism of LINC01296 in diverse cancer types, and introduced the related clinical significance. However, due to the limited sample size, the research on LINC01296 in cancers is still in the early stage, and there are still a lot of translational studies to be carried out. It is necessary to have a thorough understanding of the pathophysiological mechanism of LINC01296 before clinical application. The stable and efficient development and detection of LINC01296 need to be further studied. In the future, finding safe and efficient delivery systems and addressing challenges, such as poor specificity and off-target effects, are critical. It is hoped that this review will provide new insights into clinical development and the search for new biomarkers, and that high-quality experimental and clinical studies centered on LINC01296 will provide cancer patients with more effective treatment options and better survival prospects.

## Figures and Tables

**Fig. (3):**
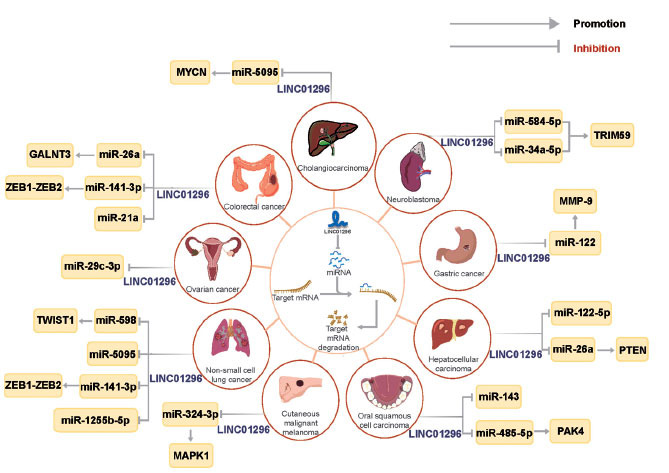
A comprehensive diagram illustrating the involvement of lncRNA-miRNA-mRNA interactions in various types of human cancers utilizing LINC01296. miRNAs have the ability to interact with the 3′-untranslated regions of protein-coding mRNAs, leading to degradation and repression of targeted mRNA translation. However, LINC01296 can counter these inhibitory effects on mRNA translation. LINC01296 exhibits ceRNA functions through different pathways, manipulating the expression of signaling molecules/pathways associated with cancer in multiple malignancies.

**Table 1:** The clinical significance of LINC01296 in various cancers.

**Cancer Type**	**Numbers of Clinical Samples**	**Expression**	**Clinical Characteristics**	**Prognostic Implication of LINC01296 Overexpression**	**Property**	**References**
Hepatocellular carcinoma	50	Upregulation	alpha fetoprotein levels, tumor size, TNM stage, and overall survival rate	Poor	Oncogene	[17]
Gastric cancer	60	Upregulation	TNM stage, tumor size, lymph node metastasis	Poor	Oncogene	[20]
Oral squamous cell carcinoma	40	Upregulation	TNM stage, nodal invasion	Poor	Oncogene	[22]
-	58	Upregulation	Low survival rate	Poor	Oncogene	[30]
Neuroblastoma	43	Upregulation	TNM stage	Poor	Oncogene	[23]
Cholangiocarcinoma	57	Upregulation	TNM stage and lymph node metastasis	Poor	Oncogene	[24]
Non-small cell lung cancer	40	Upregulation	TNM stage	Poor	Oncogene	[25]
-	40	Upregulation	TNM stage	Poor	Oncogene	[28]
-	325	Upregulation	TNM stage, Low survival rate	Poor	Oncogene	[91]
Colorectal cancer	40	Upregulation	TNM stage	Poor	Oncogene	[28]
-	51	Upregulation	TNM stage, lymph node metastasis, and distant metastasis	Poor	Oncogene	[31]
-	36	Upregulation	TNM stage, lymph node metastasis, liver metastasis and other distant metastasis	Poor	Oncogene	[38]
Prostate cancer	73	Upregulation	preoperative prostate specific antigen, lymph-node metastasis, Gleason score, TNM stage	Poor	Oncogene	[37]
Ovarian cancer	92	Upregulation	TNM stage, lymph node metastasis	Poor	Oncogene	[47]
Neuroblastoma	28	Upregulation	Tumor recurrence, progression, and death	Poor	Oncogene	[54]
Esophageal squamous cell carcinoma	221	Upregulation	differentiation grade, lymph node metastasis, distant metas tasis, and TNM stage	Poor	Oncogene	[57]
-	78	Upregulation	TNM stage, lymph node metastasis	Poor	Oncogene	[58]
Bladder cancer	78	Upregulation	TNM stage, lymph node metastasis and pathologic grades	Poor	Oncogene	[67]
Breast cancer	55	Upregulation	tumor size, lymph node metastasis, and advanced TNM stage	Poor	Oncogene	[78]
Pancreatic ductal Adenocarcinoma	85	Upregulation	TNM stage, lymph node metastasis	Poor	Oncogene	[80]
Clear cell renal cell carcinoma	80	Upregulation	TNM stage, lymph node metastasis, pathologic grades and low survival rate	Poor	Oncogene	[81]

**Table 2:** Functions and mechanisms of LINC01296 in various malignancies.

**Cancer Type**	**Cell Lines**	**Expression**	**Function**	**Regulatory Mechanism**	**Role**	**References**
Hepatocellular carcinoma	SMMC-7721, Hep3B, HepG2, and Huh7	Upregulation	migration↑, invasion↑, proliferation↑	LINC01296/miR-122-5P	Oncogene	[17]
-	Huh-7, SMMC-7721, BEL-7404, and HepG	Upregulation	migration↑, invasion↑, proliferation↑, apoptosis↓	LINC01296/miR-26a/PTEN	Oncogene	[50]
Non-small cell lung cancer	H1299, A549, PC-9, HCC827, 95-D, and H1975	Upregulation	migration↑, proliferation↑, cell cycle↑, apoptosis↓	LINC01296/miR-143-3p/ATG2B	Oncogene	[18]
-	A549, NCI-H1299, SK-MES-1, and Calu-3	Upregulation	proliferation↑, apoptosis↓	LINC01296/miR-598/Twist1	Oncogene	[25]
-	H1299, SPC-A1, A549 and H23	Upregulation	migration↑, proliferation↑	LINC01296/miR-5095	Oncogene	[26]
-	A549 and H1299	Upregulation	migration↑, invasion↑	LINC01296/miR-141-3p/ZEB1-ZEB2	Oncogene	[28]
-	H1299、A549、HCC827、H1975、PC-9 and 95-D	Upregulation	migration↑, proliferation↑	LINC01296/miR-1225b-5p	Oncogene	[71]
Gastric cancer	SGC-7901, BGC-823, MGC-803, and MKN-45	Upregulation	migration↑, invasion↑, proliferation↑, apoptosis↓	LINC01296/miR-122/MMP-9	Oncogene	[20]
Oral squamous cell carcinoma	SCC-15, CAL-27, HSC-2, Tca8113 and SCC-9	Upregulation	migration↑, invasion↑, proliferation↑, apoptosis↓	LINC01296/miR-485-5p/PAK4	Oncogene	[22]
-	HSC-2, CAL-27, and SCC-25	Upregulation	migration↑, invasion↑, proliferation↑	-	Oncogene	[77]
Neuroblastoma	SK-N-SH and IMR-32	Upregulation	migration↑, invasion↑, proliferation↑, apoptosis↓	LINC01296/miR-584-5p and miR-34a-5p/TRIM59	Oncogene	[23]
-	SK-N-BE(2)C, CHLA20, HEK293T	Upregulation	migration↑, proliferation↑, apoptosis↓	LINC01296/NCL/SOX11	Oncogene	[54]
Cholangiocarcinoma	HIBEC, RBE, CCLP1, HuCCT1, HCCC-9810	Upregulation	migration↑, invasion↑, apoptosis↓	LINC01296/ miR-5095/MYCN	Oncogene	[24]
Cutaneous malignant melanoma	A-375, M21, SK-MEL-2, and A2058	Upregulation	proliferation↑	LINC01296/miR-324-3p/MAPK1	Oncogene	[27]
Colorectal cancer	SW480 and HCT116	Upregulation	migration↑, invasion↑	LINC01296/miR-141-3p/ZEB1-ZEB2	Oncogene	[28]
-	SW480, SW620, LoVo, HT29, DLD1, and HCT116	Upregulation	migration↑, invasion↑, proliferation↑, apoptosis↓	LINC01296/p15	Oncogene	[31]
-	SW620, SW480, HCT-8, 5-FU-resistant HCT-8 cells	Upregulation	proliferation↑, metastasis↑ and chemoresistance↑	LINC01296/miR-26a/GALNT3 axis activated PI3K/AKT signaling pathway	Oncogene	[38]
Oral squamous cell carcinoma	CAL-27	Upregulation	migration↑, invasion↑, proliferation↑	LINC01296/SRSF1	Oncogene	[30]
-	SAS and GNM	Upregulation	Aggressiveness↑, migration↑, invasion↑	LINC01296/miR-143	Oncogene	[76]
Osteosarcoma	hFOB1.19, MG63 and 143B	Upregulation	migration↑, invasion↑, proliferation↑, cell cycle↑	LINC01296/cyclin D1	Oncogene	[32]
Prostate cancer	22Rv1 and LNCaP	Upregulation	migration↑, invasion↑, proliferation↑	PI3K–Akt–mTOR signaling pathway	Oncogene	[37]
Ovarian cancer	SKOV3, Caov-3, HO-8910, and OVCAR3	Upregulation	migration↑, invasion↑, proliferation↑, cell cycle↑, apoptosis↓	-	Oncogene	[46]
-	SKOV-3, OVCAR-3	Upregulation	migration↑, invasion↑, proliferation↑	LINC01296/miR-29c-3p	Oncogene	[47]
esophageal squamous cell carcinoma	ECa-109, EC-9706, TE-1	Upregulation	migration↑, invasion↑, proliferation↑	-	Oncogene	[57]
-	EC9706, E106, and TE-1	Upregulation	invasion↑, proliferation↑	LINC01296/KLF2	Oncogene	[58]
Bladder cancer	RT4, T24, 5637	Upregulation	migration↑, proliferation↑, cell cycle↑	EMT pathway	Oncogene	[67]
-	MCF7, MDA-MB-231, SKBR3, BT-20, T47D, MDA-MB-436	Upregulation	migration↑, invasion↑, proliferation↑, apoptosis↓	Bcl-2/caspase-3 signaling pathway	Oncogene	[78]
Head and neck squamous cell carcinoma	Cal-27 and SCC-9	Upregulation	migration↑, invasion↑	-	Oncogene	[79]
Pancreatic ductal adenocarcinoma	PANC-1, Capan-2, SW1990, and BxPC-3	Upregulation	migration↑, invasion↑, proliferation↑, apoptosis↓	Bcl-2/caspase-3 signaling pathway	Oncogene	[80]

**Note: ** ↑ means promoting; ↓ means inhibiting.

**Table 3:** Effects of LINC01296 on growth and metastasis of cancer xenografts.

**Cancer Type**	**Animal Models**	**Function**	**References**
Non-small cell lung cancer	6-week-old female nude mice	↓↓LINC01296: ↓ tumor growth, tumor sizes, ↓Ki-67, E-cadherin, ↑ N-cadherin	[18]
-	SPF male nude mice	↓↓LINC01296: ↓ tumor growth, tumor sizes	[25]
-	6-week-old female BALB/c nude mice	↓↓LINC01296: ↓ tumor growth, tumor migration	[71]
Gastric cancer	6-week-old male BALB/c nude mice	↓↓LINC01296: ↓ tumor growth, tumor volumes	[20]
Oral squamous cell carcinoma	6-week-old male BALB/c nude mice	↓↓LINC01296: ↓ tumor growth	[22]
Cholangiocarcinoma	6-week-old male BALB/c nude mice	↓↓LINC01296: ↓ tumor growth, tumor migration	[24]
Cutaneous malignant melanoma	4- to 6-week-old female BALB/c nude mice	↓↓LINC01296: ↓ tumor sizes, ↓Ki-67, MAPK1	[27]
Oral squamous cell carcinoma	8-week-old female BALB/c nude mice	↑↑LINC01296: ↑ tumor volumes, tumor weight, ↑ Ki-67	[30]
Colorectal cancer	4-week-old male nude mice	↓↓LINC01296: ↓ tumor growth, tumor weight, ↓Ki-67	[31]
Neuroblastoma	6-week-old male BALB/c nude mice	↑↑ LINC01296: ↑tumor volumes	[54]

**Note: ** ↑↑ LINC01296: ↑↑ means LINC01296 overexpression; ↑: ↑ means promoting. ↓↓ LINC01296: ↓↓ means LINC01296 knockdown or knockout; ↓: ↓ means inhibiting.

## LIST OF ABBREVIATIONS

LncRNAs: Long non-coding RNAs
LINC01296: Long intergenic non-coding RNA 01296
LIHC: Liver hepatocellular carcinoma
LUAD: Lung adenocarcinoma
STAD: Stomach adenocarcinoma
ceRNA: Competitive endogenous RNA
EMT: Epithelial-mesenchymal transition
OC: Ovarian cancer
HCC: Hepatocellular carcinoma
NB: Neuroblastoma
ESCC: Esophageal squamous cell carcinoma
CRC: Colorectal cancer
MUC1: Mucin1
BC: Bladder cancer
qRT-PCR: Quantitative reverse transcriptase PCR
SCLC: Small cell lung cancer
NSCLC: Non-small cell lung cancer
OSCC: Oral squamous cell carcinoma
BRCA: Breast cancer
HNSCC: Head and neck squamous cell carcinoma
GC: Gastric cancer
OS: Osteosarcoma
CCA: Cholangiocarcinoma
PDAC: Pancreatic ductal adenocarcinoma
PC: Prostate cancer
CMM: Cutaneous malignant melanoma
TNM: Tumor nodes metastases
KLF2: Kruppel-like factor 2
GALNT3: Polypeptide N-acetylgalactosaminyltransferase 3
MMP-9: Matrix metalloproteinase-9
PAK4: P21-activating kinase 4
P-ERK: P-extracellular signal-regulated kinase
TRIM59: Tripartite motif-containing 59
MAPK1: Mitogen-activated protein kinase 1
CEA: Carcinoembryonic antigen
CA19-9: Cancer antigen 19-9
ccRCC: Clear cell renal cell carcinoma
